# Modulation of Ryanodine Receptors Activity Alters the Course of Experimental Autoimmune Encephalomyelitis in Mice

**DOI:** 10.3389/fphys.2021.770820

**Published:** 2021-12-17

**Authors:** Natalia C. Osipchuk, Athena M. Soulika, Alla F. Fomina

**Affiliations:** ^1^Department of Physiology and Membrane Biology, University of California, Davis, Davis, CA, United States; ^2^Shriners Hospitals for Children Northern California, Institute for Pediatric Regenerative Research, Sacramento, CA, United States; ^3^Department of Dermatology, University of California, Davis, Davis, CA, United States

**Keywords:** ryanodine receptors, dantrolene, *RYR1-p.R163C* mutation, immunomodulation, experimental autoimmune encephalomyelitis, multiple sclerosis

## Abstract

Ryanodine receptors (RyRs), the intracellular Ca^2+^ release channels, are expressed in T lymphocytes and other types of immune cells. Modulation of RyRs has been shown to affect T cell functions *in vitro* and immune responses *in vivo*. The effects of modulation of RyRs on the development of autoimmune diseases have not been investigated. Here we studied how modulation of RyRs through administration of RyR inhibitor dantrolene or introducing a gain-of-function *RYR1*-p.R163C mutation affects clinical progression of experimental autoimmune encephalomyelitis (EAE) in mice, a T cell-mediated autoimmune neuroinflammatory disease. We found that daily intraperitoneal administration of 5 or 10 mg/kg dantrolene beginning at the time of EAE induction significantly reduced the severity of EAE clinical symptoms and dampened inflammation in the spinal cord. The protective effect of dantrolene on EAE was reversible. Dantrolene administration elicited dose-dependent skeletal muscle weakness: mice that received 10 mg/kg dose developed a waddling gait, while 5 mg/kg dantrolene dose administration produced a reduction in four-limb holding impulse values. Mice bearing the gain-of-function *RYR1-p.R163C* mutation developed the EAE clinical symptoms faster and more severely than wild-type mice. This study demonstrates that RyRs play a significant role in EAE pathogenesis and suggests that inhibition of RyRs with low doses of dantrolene may have a protective effect against autoimmunity and inflammation in humans.

## Introduction

Ryanodine receptors (RyRs) are a family of intracellular Ca^2+^ release channels located in the membranes of the smooth endoplasmic reticulum. Three different genes encode RyRs: type 1 (RyR1), type 2 (RyR2), and type 3 (RyR3) with additional transcripts produced by alternative splicing (Fill and Copello, 2002). RyRs are expressed in the immune cells of both lymphoid (Inngjerdingen et al., 1999; Girard et al., 2001; Hosoi et al., 2001; Gao et al., 2005; Schiemann et al., 2014) and myeloid (Hosoi et al., 2001; O’Connell et al., 2002) lineages. The role of RyRs in immune cell Ca^2+^ signaling is best understood in T lymphocytes, in which RyRs regulate Ca^2+^ release from the intracellular store and, subsequently, store-operated Ca^2+^ entry (Dadsetan et al., 2008; Thakur et al., 2012; Fomina, 2021). The expression of different types of RyRs depends on the environmental factors and functional state of T cells. Cytokines and T cell receptor agonists induce expression of RyR1 and RyR2, of which the former appears to be a predominant isoform in activated T cells (Hosoi et al., 2001; Thakur et al., 2012).

Because intracellular Ca^2+^ dynamics regulate primary T cell functions, including motility, proliferation, and cytokine production (Trebak and Kinet, 2019; Vaeth et al., 2020), modulation of RyRs either by gain-of-function mutations, genetic deletion, or pharmacological inhibition has been shown to alter T cell intracellular Ca^2+^ signaling and immunological responses *in vitro* and *in vivo* (Fomina, 2021). In *in vitro* experiments on human and mouse T cells, an inhibitor of RyR1 and RyR3 dantrolene (Zhao et al., 2001; Krause et al., 2004) has been found to suppress the Ca^2+^ signaling, proliferation, interleukin-2 production, and interfered with the interleukin-2 -receptor signaling (Conrad et al., 2004; Thakur et al., 2012). In mice challenged with lipopolysaccharide to induce an inflammatory response, dantrolene administration reduced plasma concentration of the pro-inflammatory cytokines tumor necrosis factor-α, interferon-γ, and interleukin-12, while elevated the level of the anti-inflammatory cytokine interleukin-10 (Hasko et al., 1998; Nemeth et al., 1998). T cells derived from knock-in mice heterozygous for the human N-terminal gain-of-function mutation *RYR1*-p.R163C (referred to as R163C HET mice here) (Yang et al., 2006) displayed leaky intracellular Ca^2+^ store, elevated intracellular Ca^2+^ concentration at rest, altered mitochondrial function, and earlier onset of the stationary phase of *in vitro* growth compared with wild-type (WT) T cells (Yang et al., 2021). Treatment of T cells derived from the R163C HET mice with dantrolene restored the cytosolic Ca^2+^ levels and Ca^2+^ store content, indicating that aberrations in T cell Ca^2+^ signaling were mediated by increased Ca^2+^ discharge from the intracellular store via RyR1. Mice expressing another gain-of-function *RYR1*-p.Y522S mutation exhibited increased surface expression of the maturation marker CD83 on dendritic cells, elevation in the total number of circulating lymphocytes and the number of splenic CD4^+^ T cells, higher levels of circulating natural antibodies (IgG_1_ and IgE), and more efficient specific immune response following an antigenic challenge with ovalbumin or a helminthic parasite *Heligmosomoides polygyrus bakeri*, compared with WT mice (Vukcevic et al., 2013). Collectively, these data indicate that RyRs regulate T cell Ca^2+^ signaling and Ca^2+^-dependent functions *in vitro* and affect expression of the resting immune markers and adaptive immune response to pathogens or endotoxins *in vivo.*

Multiple sclerosis (MS) is a neuroinflammatory auto-immune disease of the central nervous system, mediated by myelin-reactive T cells (Haase and Linker, 2021). Previous studies showed that inhibiting T cell plasmalemmal store-operated channels alleviated disease severity in the mouse model of MS, experimental autoimmune encephalomyelitis (EAE) (Park et al., 2020; Vaeth et al., 2020), indicating that suppression of Ca^2+^ signaling in T cells may represent a new strategy for treating MS and other T cell-mediated inflammatory or autoimmune diseases. At present, several store-operated channel inhibitors are under investigation in clinical trials (Stauderman, 2018; Waldron et al., 2019; Khan et al., 2020). However, their safety and efficacy in treating autoimmune diseases in humans are yet to be determined. Because RyRs control T cell store-operated channel gating by regulating Ca^2+^ release from the store, the question arises if it would be possible to alter the course of autoimmune disease through modulation of RyRs activity. The RyR inhibitor dantrolene is approved by the FDA for treating diseases associated with skeletal muscle dysfunctions, such as malignant hyperthermia syndrome, spasticity, and core diseases (Krause et al., 2004). If suppression of RyRs alleviates T cell-mediated autoimmune diseases, then it would be possible to repurpose dantrolene for treating these diseases. Here we investigated the effect of dantrolene administration on clinical progression of EAE in mice. To confirm the involvement of RyRs in EAE pathogenesis, we also studied the impact of RyR1 gain-of-function mutation on EAE progression using the R163C HET mice.

## Methods

### Animals

All animal experiments were conducted using protocols approved by the Institutional Animal Care and Use Committees at the University of California, Davis. C57BL/6 male mice were purchased from Jackson Laboratories. R163C HET mice were generously provided by Dr. P.D. Allen. Generation and genotyping of R163C HET mice were performed as previously described (Yang et al., 2006). Mice were housed in climate-controlled rooms and maintained on standard mouse chow and water supply.

### Experimental Autoimmune Encephalomyelitis Induction, Treatment, and Scoring

EAE was induced in mice by subcutaneous administration of a total of 300 μg in the flanks (each flank received 150 μg) of rodent myelin oligodendrocyte glycoprotein peptide 35–55 (MEVGWYRSPFSRVVHLYRNGK, New England Peptide) emulsified in the complete Freund’s adjuvant containing *Mycobacterium tuberculosis* H37Ra (Difco). *Bordetella pertussis* toxin (200 ng; List Biological Laboratories) was administered intraperitoneally (i.p.) on the day of immunization and 48 h after (Soulika et al., 2009; Lee et al., 2012). To study the effects of pharmacological inhibition of RyRs with dantrolene, EAE was induced in age-matched 10–12 week-old male C57BL/6 mice; to study the effects of *RYR1*-p.R163C mutation, the EAE was induced in sex-matched 10–17 week-old R163C HET mice and their WT littermates of both sexes.

For dantrolene experiments, mice were randomly assigned into two groups: mice injected with complete Freund’s adjuvant alone (control mice) and mice with induced EAE (EAE mice). Animals in both EAE and control groups were further assigned to one of the five subgroups, which received daily injections of different doses of dantrolene, as shown in Table 1. Mice received equal volumes of the vehicle or dantrolene-containing solutions (20–30 μl per 20–30 g mouse or 1 μl per 1 g of weight). Dantrolene or vehicle were delivered via i.p. injections starting at the time of EAE induction [day 0 post-immunization (p.i.)] and were administered daily during the experiment’s duration unless indicated otherwise.

**TABLE 1:** EAE and control mice dantrolene treatment group assignment.

Group name	EAE induction	Dantrolene concentration	Number of mice**
EAE + Veh	Yes	0*	9
EAE + 5 Dan	Yes	5 mg/kg	8
EAE + 10 Dan	Yes	10 mg/kg	8
5 Dan	No	5 mg/kg	5
10 Dan	No	10 mg/kg	5

*Mice were injected with vehicle composed of the miglyol 812/ethanol (90/10 v/v mixture). **The number of mice at the beginning of the experiment. One mouse in the 5 Dan group developed paraphimosis and was excluded from the experiment after day 15. One mouse in the 10 Dan groups died on day 24. The data pertinent to the 5 Dan and 10 Dan groups after day 15 and day 24, respectively, are obtained from 4 remaining mice in each group.

Mice in all groups were weighed and scored for EAE symptoms daily by an experienced investigator blinded to the treatment received or the genetic background of each mouse. In R163C HET mice and their WT littermates, the rectal temperature was monitored by a thermistor. Clinical scores were assigned as described previously (Soulika et al., 2009) with modifications: 0.0, no noticeable signs in motor disfunction; 0.5, distal limp tail or light waddling gait; 1.0, limp tail or waddling gait (dragging of the hindlimbs); 1.5, limp tail and waddling gait; 2, single limb paresis; 3, double limb paresis; 3.25, severe double limb paresis; 3.5, single-limb paralysis and paresis of the second limb; 4, paralysis of 2 limbs; 4.5, moribund; 5, death.

For dantrolene injections, dantrolene sodium (MilliporeSigma) was converted to dantrolene (Supplementary Material 1) to improve solubility in the injection vehicle composed of a 90/10 v/v mixture of miglyol 812 (Medlab International) and ethanol (200 proof; Fisher Sci.). Dantrolene was reconstituted in the vehicle preheated to 40°C at a concentration of 8 mg/ml and stirred until the solution became clear. Dantrolene stock solution was aliquoted into glass scintillation vials and stored at −20°C. Before administration to the animals, the stock solution of dantrolene was thawed and preheated to 40°C to ensure that dantrolene stays in solution and then cooled down to room temperature.

### Spinal Cord Immunohistology

Mice were sacrificed on days 38 or 42 p.i. and sections of the lumbar spinal cord were prepared for immunohistochemistry as previously described (Lee et al., 2012). Briefly, spinal cords were collected after whole-body perfusion with ice-cold PBS and then post-fixed in 4% paraformaldehyde (Electron Microscopy Sciences) in PBS for 24 h, then moved to 30% sucrose (Thermo Fisher Scientific) in PBS for 72 h. Spinal cords were embedded in cryostat mounting media (Tissue-Tek OCT, Sakura Finetek). Ten-micrometer sections were dried at room temperature for 1 h, rinsed with PBS, and blocked with 10% donkey serum or goat serum (Jackson ImmunoResearch), depending on the secondary antibody used. Primary antibodies were applied overnight at 4°C and were rat anti-mouse CD4 (BD Pharmingen, 1:100), rabbit polyclonal Iba-1 (Wako Chemicals, 1:500). Fluorescently conjugated secondary antibodies to DyLight488™ or DyLight549™ (Jackson ImmunoResearch, 1:500) or AlexaFluor-488, AlexaFluor-549, or Cyanine Cy5 (Jackson ImmunoResearch, 1:500) for 1 h at room temperature. Nuclei were counter-stained with DAPI. Images were obtained by laser scanning confocal microscopy with 20 × objectives (Nikon C2) and analyzed using the Nikon NIS-Elements software (Version 5.02).

### Non-invasive Skeletal Muscle Function Test

Two groups of 14-week-old male C57BL/6 mice were used: (1) mice receiving the daily i.p. injections of 5 mg/kg dantrolene and (2) littermate control mice receiving the daily i.p. injections of the vehicle alone [miglyol 812/ethanol (90/10 v/v mixture)]. A four-limb inverted wire grid hanging test was performed as previously described (Carlson et al., 2010; Aartsma-Rus and van Putten, 2014; Bonetto et al., 2015). Briefly, the mouse was placed on the top and at the center of the custom-made 26 × 20 cm wire grid (1.2 × 1.2 cm square grid; 1 mm wire diameter) attached to the wide wood frame, which prevented mice from escaping the grid. The structure was then inverted and suspended at the heights of 20–30 cm above the soft bedding. The time between the frame inversion and the animal falls off the grid (“hang time”) was recorded with a stopwatch. To account for the effects of body weight on the hanging time, each mouse body weight was recorded shortly before the four-limb hanging test and used to calculate the holding impulse, a product of the hang time and body weight (s × g). The same operator performed the test at the exact location and at the same time of the day.

### Statistical Analysis

One-way ANOVA, two-way repeated-measures ANOVA, Friedman ANOVA, or Student’s *t*-test (Origin 7 and OriginPro software; Origin Lab) were used as appropriate. Bonferroni correction was applied for multiple comparisons. *P* < 0.05 was considered statistically significant.

## Results

### Administration of Dantrolene Alleviates Experimental Autoimmune Encephalomyelitis Symptoms and Reduces Neuroinflammation

**FIGURE 1:**
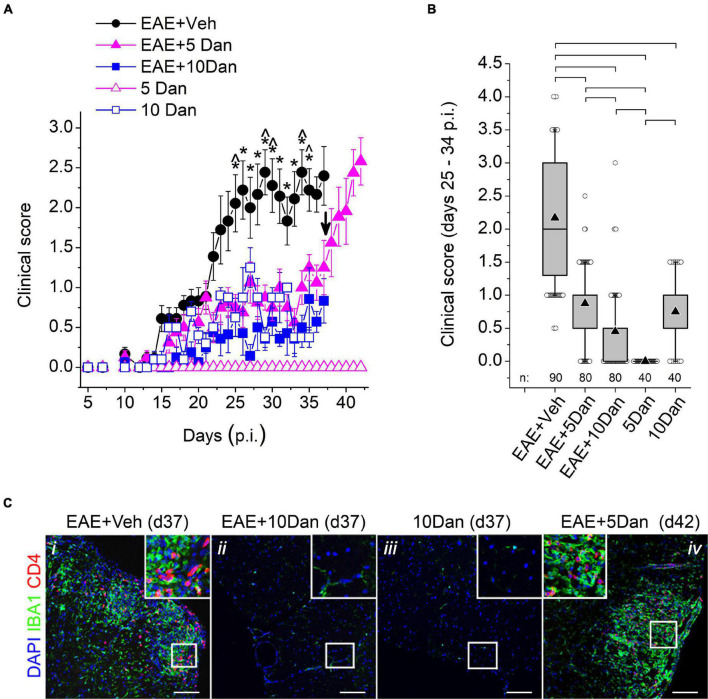
Dantrolene alleviates clinical symptoms and reduces CNS inflammation in EAE mice. **(A)** Daily EAE clinical scores of mice injected with vehicle (EAE + Veh; *n* = 9), 5 mg/kg dantrolene (EAE + 5 Dan; *n* = 8), 10 mg/kg dantrolene (EAE + 10 Dan; *n* = 8), and control groups mice in which EAE was not induced injected with 5 mg/kg dantrolene (5 Dan; *n* = 5 before day 16 and *n* = 4 on and after day 16), or 10 mg/kg dantrolene (10 Dan; *n* = 5 before day 24 and *n* = 4 on and after day 25). Injections of vehicle or dantrolene solutions began at the time of immunization and were delivered daily after that. Data were collected during one experiment and are shown as mean ± SE; n, number of animals in the group. Asterisks denote significant difference between mean scores in the EAE + Veh group and mean scores in EAE + 5 Dan; EAE + 10 Dan, and 5 Dan groups; circumflexes denote significant difference between average scores in the EAE + Veh group and those in all other groups (two-way repeated-measures ANOVA with Bonferroni correction; *p* < 0.05). Beginning on day 37 p.i., mice in the EAE + 5 Dan group were injected with vehicle alone (indicated with an arrow). Note the rapid increase in clinical scores after cessation of administration of 5 mg/kg dantrolene. **(B)** Box charts of clinical scores recorded from day 25 p.i. until day 34 p.i. in five treatment groups. Data are from the same treatment groups shown in **(A)**. Brackets indicate that differences between treatment group means are significant (*p* < 0.01; one-way ANOVA with Bonferroni correction). In the box charts, the boundary of the box closest to zero indicates the 25th percentile, a horizontal line within the box marks the median, a black up-pointing triangle within the box marks the mean, and the boundary of the box farthest from zero indicates the 75th percentile. Whiskers above and below the box indicate the 10th and 90th percentiles. Open circles above and below the whiskers indicate outliers outside the 10th and 90th percentiles. The total combined number of measurements per treatment group (n) are shown at the bottom of the graph. **(C)** Spinal cord sections stained for IBA^+^ and CD4^+^ infiltrating cells obtained on day 37 p.i. from EAE mouse injected daily with vehicle (EAE + Veh; panel *i*), EAE mouse injected daily with 10 mg/kg dantrolene (EAE + 10 Dan; panel *ii*), and control mouse injected with 10 mg/kg dantrolene (10 Dan; panel *iii*). Right panel (EAE + 5 Dan; panel *iv*) shows spinal cord section obtained on day 45 p.i. from EAE mice injected daily with 5 mg/kg dantrolene from day 0 to day 37 p.i. and with vehicle alone after that. IBA1 is a marker for microglia and infiltrating macrophages; CD4 is a marker for infiltrated CD4^+^ T cells; nuclei were contrasted with DAPI. Representative images from spinal cord sections from four randomly selected mice, which were examined for each condition. Scale bars, 100 μm. Boxed area at the top of each panel is an expended boxed area shown in the middle of the panel. Each boxed area is 100 × 100 μm.

Figure 1A shows EAE clinical course in five experimental groups listed in Table 1. The EAE mice injected with vehicle alone (EAE + Veh group) displayed an increase in clinical scores after the 10–16-day latent period and reached the maximal score values between day 23 and 28 p.i., after which the mice assumed a chronic, persistent EAE disability phenotype. The mice injected with 5 or 10 mg/kg dantrolene (EAE + 5 Dan and EAE + 10 Dan groups) displayed significantly less severe EAE clinical symptoms than the mice in the EAE + Veh group (Figure 1A and Supplementary Materials 2–4). The average EAE clinical score values recorded on days 25–34 p.i. in the EAE + 5 Dan and EAE + 10 Dan groups were 60 and 80% smaller, respectively, than those in the EAE + Veh group (Figure 1B). Control mice in the 10 Dan group developed a waddling gait and reduced tail tone and were assigned EAE clinical scores between 0.5 and 1.5 (Figures 1A,B; 10 Dan group). Control mice in the 5 Dan group did not develop noticeable movement deficits assigned to the EAE clinical score values (Supplementary Materials 5, 6). Mice from the EAE + Veh, EAE + 10 Dan and 10 Dan groups were sacrificed on day 37 p.i. and spinal cords sections from these mice were immunostained for infiltrating CD4^+^ T cells and microglia/macrophages. The inflammatory infiltrate was present in the spinal cords of mice from the EAE + Veh group, but not from EAE + 10 Dan and 10 Dan groups (Figure 1C). Beginning on day 37 p.i., mice in the EAE + 5 Dan group were injected daily with vehicle alone. After dantrolene withdrawal, mice in the EAE + 5 Dan group rapidly developed neurological deficits (Figure 1A), which were associated with spinal cord infiltration detected on day 42 p.i. (Figure 1C).

### Administration of 5 mg/kg Dantrolene Causes Skeletal Muscle Weakness

**FIGURE 2:**
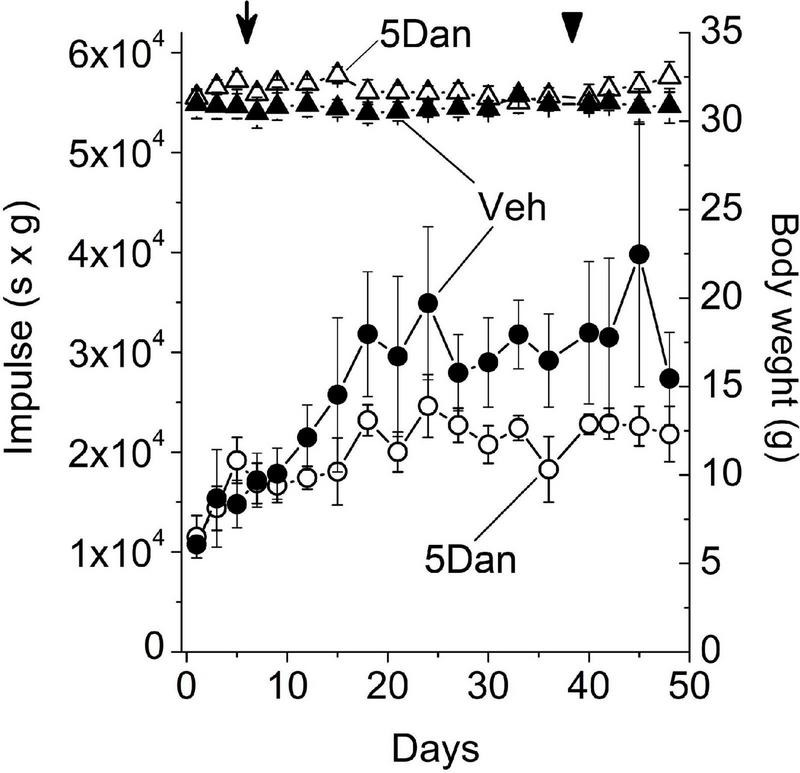
Administration of dantrolene reduces four-limb holding impulse. Holding impulse (hang time × body weight; left axis) in healthy mice injected daily with vehicle (closed circles; *n* = 4) or 5 mg/kg dantrolene (open circles; *n* = 4). Injections of both vehicle and dantrolene solution began on day 3 after the beginning of the experiment (arrow) and were administered daily until day 36. Starting from day 37 (arrowhead), the mice in both groups were injected daily with saline. Friedman ANOVA analyses showed that mice injected with 5 mg/kg dantrolene tend to have smaller holding impulse values than mice injected with vehicle alone (*p* < 0.05). Closed and open triangles show the body weight of mice (right axis) injected with vehicle (closed triangles; *n* = 4) or 5 mg/kg dantrolene (open triangles; *n* = 4). Data are shown as mean ± SE. n, the number of mice in each group. Data are representative of one experiment.

The relaxing effect of dantrolene on skeletal muscle is well established (Herman et al., 1972; Ellis and Carpenter, 1974; Ellis et al., 1976). Therefore, movement deficits observed in the control mice injected with 10 mg/kg dantrolene (Figures 1A,B; 10 Dan group) in the absence of inflammatory infiltrate in the CNS (Figure 1C) are likely caused by an adverse effect of dantrolene on skeletal muscle. Given that administration of 5 mg/kg dantrolene did not display noticeable upright movement deficits on flat-surface (Figures 1A,B; 5 Dan group), we explored whether this dose of dantrolene exerts an adverse effect on the skeletal muscle function using an inverted wire grid four-limb hang test. This test measures the ability of the mice to remain clinging to an inverted wire grid and evaluates general neuromuscular conditions over time (Carlson et al., 2010). All mice were trained on the inverted grid for 3 days before the beginning of the experiment, after which all mice were willing to cling to the grid without intentionally jumping off of the grid (Supplementary Materials 7, 8). Mice hanging time and body weight were recorded for 3 days before beginning administration of 5 mg/kg dantrolene or vehicle and were comparable in both experimental groups (Figure 2). In both groups, mice maintained a stable body weight during the entire experiment. Holding impulse (hang time × body weight) increased in both groups of mice from day 0 through day 18, presumably due to the learning effect on repeated trials. After this period, the average impulse values remained relatively constant throughout the experiment in each group. Despite the considerable variability in holding impulse values among mice in each group, statistical analysis revealed that mice injected with 5 mg/kg dantrolene tend to have smaller holding impulse values compared with mice injected with vehicle alone (*p* < 0.05; Friedman ANOVA), indicating that continuous administration of 5 mg/kg dantrolene elicited skeletal muscle function deficit. Replacement of vehicle and 5 mg/kg dantrolene solutions with physiological saline at the end of the experiment did not improve the performance on the inverted grid within 10 days after cessation of dantrolene administration.

### R163C HET Mice Display Accelerated Development of Experimental Autoimmune Encephalomyelitis Symptoms

**FIGURE 3:**
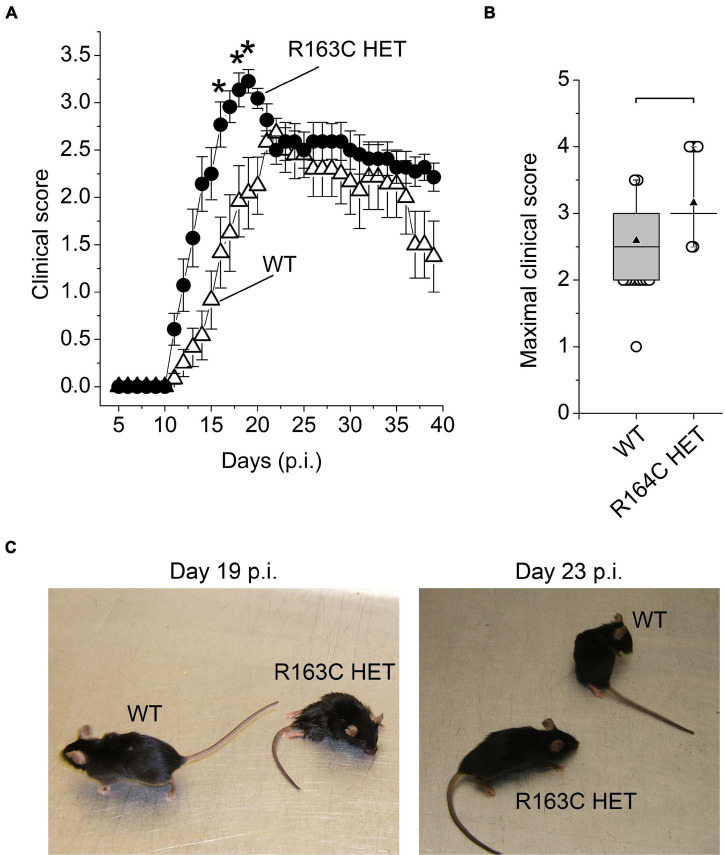
A *RyR1* gain-of-function mutation accelerates development of EAE symptoms. **(A)** Cumulative clinical scores in WT (open circles; *n* = 12) and R163C HET (black circles; *n* = 12) mice. n, number of animals in the group. EAE was induced in both groups of mice on the same day (day 0). Asterisks (*) denote significant differences between mean scores in WT and R163C HET groups (two-way repeated-measures ANOVA with Bonferroni correction; *p* < 0.05). Combined data from two experiments (6 mice per group in each experiment) shown as mean ± SE. **(B)** Box charts of maximal clinical scores recorded from the day 14 p.i. until day 16 p.i. in R163C HET group (*n* = 33) and from day 21 to day 23 in WT group (*n* = 33). n, number of measurements in each group. Data are from the same groups shown in **(A)**. Brackets indicate that differences between means are significant (*p* < 0.01; independent Student’s *t*-test). In the box charts, the boundary of the box closest to zero indicates the 25th percentile, a horizontal line within the box marks the median, a black up-pointing triangle within the box marks the mean, and the boundary of the box farthest from zero indicates the 75th percentile. Whiskers above and below the box indicate the 5th and 95th percentiles. Open circles above and below the whiskers indicate outliers outside the 5th and 95th percentiles. **(C)** Images of representative WT and R163C HET littermate mice at day 19 p.i. and day 23 p.i. On day 19 p.i., the clinical scores were 0.5 and 4 for WT and R163C mice, respectively; on day 23 p.i. the clinical scores were 2.5 and 3.5 for WT and R163C HET mice, respectively.

The R163 HET mice developed EAE clinical symptoms more rapidly than the WT mice (Figures 3A,C and Supplementary Materials 9, 10). On average, the maximal clinical score values were recorded on day 19 p.i. and day 22 p.i. in R163C HET mice and WT mice, respectively. The average maximal score was about 16% higher in R163 HET mice than WT mice (Figure 3B). After reaching peak values, clinical scores in the R163 HET mice group declined slightly to the levels that, on average, were comparable with the average clinical scores recorded in WT mice (Figure 3A). R163 HET mice develop fulminant malignant hyperthermia syndrome under triggering conditions, which manifests by the increased body temperature (Yang et al., 2006). To monitor the potential development of malignant hyperthermia, rectal temperatures were recorded daily starting 3 days before the EAE induction in WT and R163 HET mice. Rectal temperatures ranged from 32° to 35°C during the entire experiment, and there were no significant differences between WT and R163 HET groups of mice.

## Discussion

EAE is an established animal model of T cell-mediated autoimmune disease, which recapitulates many features of neuroinflammation and axonal damage observed in MS in humans (Constantinescu et al., 2011; Glatigny and Bettelli, 2018; Lassmann, 2020; Martinez and Peplow, 2020). EAE in mice has been widely used to examine the preclinical efficacy of immunomodulatory drugs developed for treating MS (Steinman and Zamvil, 2006; Jones et al., 2008). The present study demonstrated that RyRs play an essential role in the EAE development in mice and suggests that suppressing the RyRs activity with dantrolene could be used to attenuate autoimmune and inflammatory responses in humans. These assertions are based on the following findings: (1) mice bearing the gain-of-function *RYR1*-p.R163C mutation develop EAE clinical symptoms faster and initially they were more severe compared with those in WT mice; (2) i.p. administration of 5 or 10 mg/kg dantrolene beginning at the time of EAE induction and daily after that significantly attenuated the development of EAE clinical symptoms and inflammation in the spinal cord. Lack of T cell infiltrates in the EAE-induced dantrolene-treated group suggests that dantrolene dampened the activation and/or migration of T cells in the periphery. As expected, upon dantrolene withdrawal, EAE clinical symptoms developed rapidly.

Because RyRs are expressed in a variety of immune cells (Inngjerdingen et al., 1999; Girard et al., 2001; Hosoi et al., 2001; O’Connell et al., 2002; Gao et al., 2005; Schiemann et al., 2014), both gain-of-function mutation in RyR1 and administration of dantrolene should affect functions of several types of immune cells, which may contribute to the EAE development (Haase and Linker, 2021; Ramaglia et al., 2021). Nerveless, because myelin-reactive CD4^+^ T cells are the major contributors to EAE pathogenesis (Constantinescu and Gran, 2014; Haase and Linker, 2021; Ramaglia et al., 2021), the up- or downregulation of T cell RyRs before or at the time of the EAE induction are likely to be responsible for augmentation and alleviation, respectively, of the EAE symptoms observed in this study.

An anticipated side effect of dantrolene administration was skeletal muscle weakness in control mice, which manifested in mice developing the waddling gait and weak tail (10 mg/kg dose) or having a weaker grip (5 mg/kg dose) compared with mice injected with vehicle alone. These findings are consistent with previous reports showing that intravenous (IV) administration of dantrolene causes dose-dependent skeletal muscle weakness in humans (Flewellen et al., 1983). Although administration of 5 mg/kg dantrolene alleviated EAE symptoms to a lesser extent than 10 mg/kg dantrolene dose, EAE mice that received the 5 mg/kg dose displayed less severe skeletal muscle weakness and clinical scores below the level that reflects the presence of irreversible axon injury (<2.5) (Wujek et al., 2002). Observations that control mice injected with 5 mg/kg dose of dantrolene did not display noticeable flat-surface upright movement deficits and maintained a stable body weight for the duration of the experiment, indicate that it is possible to significantly alleviate EAE symptoms with a low dose of dantrolene without eliciting major motor deficits.

Using a dose translation from mice to humans equation (Reagan-Shaw et al., 2008), the doses of 5 and 10 mg/kg in mice are translated to equivalent doses of 0.4 and 0.8 mg/kg, respectively, in humans. From the relationship between doses of dantrolene administered IV to healthy adult volunteers and resultant peak blood concentration of dantrolene (Flewellen et al., 1983), we estimated that 0.4 and 0.8 mg/kg dose delivered via IV would result in the blood concentrations of 0.2 and 1.2 μg/ml dantrolene, respectively. A daily dose of 100 mg dantrolene administered orally to adult volunteers resulted in the peak blood levels ranging from 0.7 to 1.2 μg/ml, whereas a 50 mg daily dose of oral dantrolene resulted in 0.4 μg/ml blood level (Meyler et al., 1981). Thus, 0.4 and 0.8 mg/kg human doses of dantrolene delivered IV are estimated to be translated to daily doses of oral dantrolene below 100 mg, which are at the lower range of oral dantrolene doses indicated for treating spasticity symptoms in humans (Ratto and Joyner, 2021). Several studies reported that daily oral doses of dantrolene at or below 100 mg could be administered for decades without significant adverse effects in humans (Butala et al., 2016; Bokhari et al., 2018). These estimations cautiously suggest that continuously administered low doses of oral dantrolene (<100 mg daily) may produce a protective effect in MS or other T cell-mediated autoimmune and inflammatory diseases (e.g., psoriasis, type 1 diabetes mellitus) without significant side effects.

Dantrolene is clinically used to treat malignant hyperthermia and as a skeletal muscle relaxant to treat spasticity (Krause et al., 2004). Oral dantrolene is indicated for alleviating spasticity in MS patients (Otero-Romero et al., 2016; Patejdl and Zettl, 2017). However, dantrolene reduces spasticity at higher doses (up to 1,600 mg daily), which may cause prominent side effects, and therefore is recommended as a second-line treatment option for spasticity in MS (Otero-Romero et al., 2016). Given that all currently available disease-modifying therapies for MS differ in efficacy and each of them elicits different adverse effects (McGinley et al., 2021), expanding the application of low dose oral dantrolene to immunomodulation may increase options for the personalized combination treatment of MS and other autoimmune and inflammatory diseases in humans.

Our study demonstrated that greater degree of EAE symptoms in R163C HET mice was significant only at the early stage of the disease, whereas there were no significant differences in the mean clinical scores observed between R163C HET and WT mice at the later stages of the disease. A previous study reported that R163C HET mice had an elevated rectal temperature compared with WT mice (by 2°C on average), which might indicate that the R163C HET mice have a higher metabolic rate than WT mice (Yang et al., 2006). However, in the present study, we did not detect a significant difference in rectal temperatures between WT and R163C HET mice. The nature of the discrepancy between the results obtained in the previous study and ours is not clear. Nevertheless, our data support the conclusion of a previous study that there is no dramatic difference in the metabolic rates between WT and R163C HET mice (Yang et al., 2006).

Our previous *in vitro* studies showed that T cells derived from the R163C HET mice displayed elevated resting levels of cytosolic Ca^2+^ concentration, altered Ca^2+^ mobilization from the intracellular store and mitochondrial function, and suppressed T cell expansion in response to stimulation with a mitogenic lectin phytohemagglutinin P, compared with WT T cells (Yang et al., 2021). Elevated levels of cytosolic Ca^2+^ stimulate functions of the effector T cell subtypes believed to play a role in both EAE pathogenesis (Th17 cells) and protection (regulatory T cells) (Vaeth et al., 2020). In addition, cytosolic Ca^2+^ also causes T cell apoptosis. Thus, elevated levels of cytosolic Ca^2+^ may elicit both an accelerated rate of EAE symptoms development in R163C HET mice (e.g., due to stimulation of T cell expansion and differentiation toward inflammatory phenotypes) and a decline in the severity of the EAE symptoms at a later stage of the disease (e.g., due to expansion of the pool of protective regulatory T cells and accelerated apoptosis of pathogenic Th17 cells). Stimulatory and inhibitory effects of the gain-of-function *RYR1*-p.R163C mutation in EAE may explain an absence of association of this mutation with inflammatory and autoimmune diseases in humans.

Although previous studies suggest that animal model studies have some limitations and cannot always be extrapolated to humans (Lassmann, 2020), the present study provides proof-of-principle that RyRs represent a druggable target for treating autoimmune and inflammatory diseases in humans. Our study demonstrated efficacy of dantrolene in preventing but not treating the progressive form of the EAE. Future studies should be directed toward evaluating the efficacy of low doses of dantrolene in treating the progressive model of the EAE, as well as treating and preventing relapses in a remitting-relapsing EAE model, which resembles the most common form of MS in humans. Further studies are necessary to establish an expression profile of different isoforms of RyRs in different subsets of immune cells and whether oral dantrolene effectively alleviates inflammatory responses in humans.

## Data Availability Statement

The original contributions presented in the study are included in the article/Supplementary Material, further inquiries can be directed to the corresponding author.

## Ethics Statement

The animal study was reviewed and approved by the UC Davis IACUC.

## Author Contributions

AFF and AMS designed the research, analyzed the data, and contributed to the writing of the manuscript, and contributed equally to this work. NCO and AFF performed the experiments and data analysis. All authors contributed to the article and approved the submitted version.

## Conflict of Interest

AFF was listed as an Inventor on the U.S. Patent US8664251B1; assignee: The Regents of the University of California. The patent was related to the data presented in Figure 1 of this manuscript. The remaining authors declare that the research was conducted in the absence of any commercial or financial relationships that could be construed as a potential conflict of interest.

## Publisher’s Note

All claims expressed in this article are solely those of the authors and do not necessarily represent those of their affiliated organizations, or those of the publisher, the editors and the reviewers. Any product that may be evaluated in this article, or claim that may be made by its manufacturer, is not guaranteed or endorsed by the publisher.

## Abbreviations

RyRs: ryanodine receptors
RyR1: ryanodine receptor type 1
RyR2: ryanodine receptor type 2
RyR3: ryanodine receptor type 3
R163C HET mice: knock-in mice heterozygous for the human N-terminal mutation RYR1-p.R163C
WT: wild-type
EAE: experimental autoimmune encephalomyelitis
MS: multiple sclerosis
p.i.: post-immunization
i.p.: intraperitoneally
IV: intravenous.
