# Supplementary material for: Modulation of Ryanodine Receptors Activity Alters the Course of Experimental Autoimmune Encephalomyelitis in Mice
Source: Front Physiol. 2021 Dec 17;12:770820. doi: 10.3389/fphys.2021.770820 (PMC8751758; doi:10.3389/fphys.2021.770820)
Supplement: Supplementary file 1 [file Data_Sheet_1.docx]

**Supplementary Materials**

**Supplementary Materia 1. Method of conversion of dantrolene sodium to dantrolene (free acid).**

Dantrolene sodium (1.35 g (4 mmol); Millipore Sigma; cat # D9175) was suspended in water (20 mL) and stirred thoroughly for 30 min on a magnetic stirrer. An aqueous hydrochloric acid (4N, 4 mL, excess) was added to the resulting vermilion-colored suspension, after which the mixture was stirred thoroughly for 10 min. During this time, the vermilion-colored suspension changed into a more refined and slightly lighter suspension. The suspended solid was filtered, washed in water (4 x 15 ml), and dried, yielding a yellow solid (1.02 g, 81 %) with a melting point in the range of 278-279 °C, which is consistent with the meting point values reported for dantrolene free acid (279-280 °C) (Hosoya, Aoyama et al. 2003). The 1H and 13C NMR spectroscopy results were in agreement with those previously described for dantrolene (free acid) (Snyder, Davis et al. 1967), with GS purity above 96.6%.

**References**

Hosoya, T., H. Aoyama, T. Ikemoto, Y. Kihara, T. Hiramatsu, M. Endo and M. Suzuki (2003). "Dantrolene analogues revisited: general synthesis and specific functions capable of discriminating two kinds of Ca2+ release from sarcoplasmic reticulum of mouse skeletal muscle." Bioorg Med Chem **11**(5): 663-673.

Snyder, H. R., Jr., C. S. Davis, R. K. Bickerton and R. P. Halliday (1967). "1-[(5-arylfurfurylidene)amino]hydantoins. A new class of muscle relaxants." J Med Chem **10** (5): 807-810.

**Supplementary material 2. Video Caption:** A Representative mouse from the EAE + Veh group on day 25 p.i.

**Supplementary material 3. Video Caption:** A Representative mouse from the EAE + 5Dan group on day 26 p.i.

**Supplementary material 4. Video Caption:** A Representative mouse from the EAE + 10Dan group on day 26 p.i.

**Supplementary material 5. Video Caption:**  A Representative mouse from the 5Dan group on day 30 p.i.

**Supplementary material 6. Video Caption:** Representative mice from the 10Dan group on day 30 p.i.

**Supplementary material 7. Video Caption:** Four-limb hanging test. At the beginning of the trial, the mouse is hanging on a grid with four limbs.

**Supplementary material 8. Video Caption:** Four-limb hanging test. At the end of the trial, the mouse displays hind limbs weakness and eventually falls off the grid.

**Supplementary material 9. Video Caption:**  The EAE WT (score 0.5) and EAE R163C HET (score 4) mice on day 19 p.i

**Supplementary material 10. Video Caption.** The EAE WT (score 2.5) and EAE R163C HET (score 3.5) mice on day 23 p.i. Same mice as shown in Supplementary Material 9.
